# Supplementary figures and images for: Dynamical analysis of financial stocks network: Improving forecasting using network properties
Source: PLoS One. 2025 May 9;20(5):e0319985. doi: 10.1371/journal.pone.0319985 (PMC12063834; doi:10.1371/journal.pone.0319985)

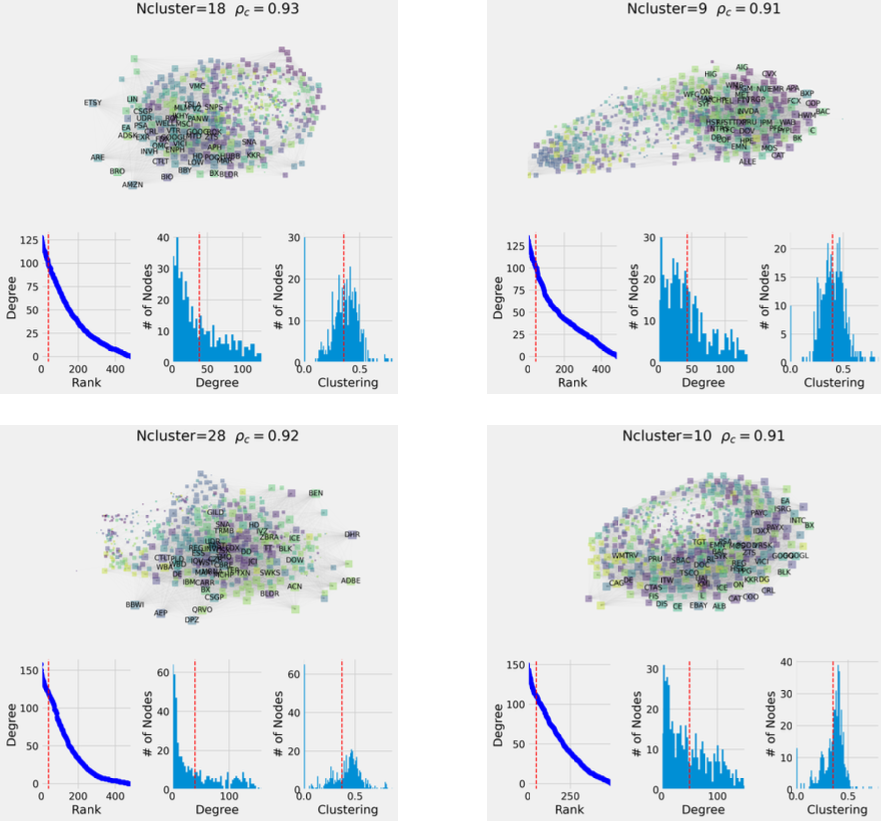

Supplement: S1 Fig — Network of the stocks for different years and their properties for the short time scale. In this figure, we display the network built on the short time scale for 4 randomly selected scale [46,106,151,211] (top panels), along with the degree distribution (left bottom panels), eigenvector centrality histogram (middle bottom panels) and clustering histogram of the stocks (right bottom panels). Red vertical lines correspond to the mean. Compared to Fig 1 the number of stocks are higher. We observe some clustering per sector (shown by the colors). In average the number of clusters found with the Louvain is smaller than in the long time period. (TIF) [file pone.0319985.s001.tif]

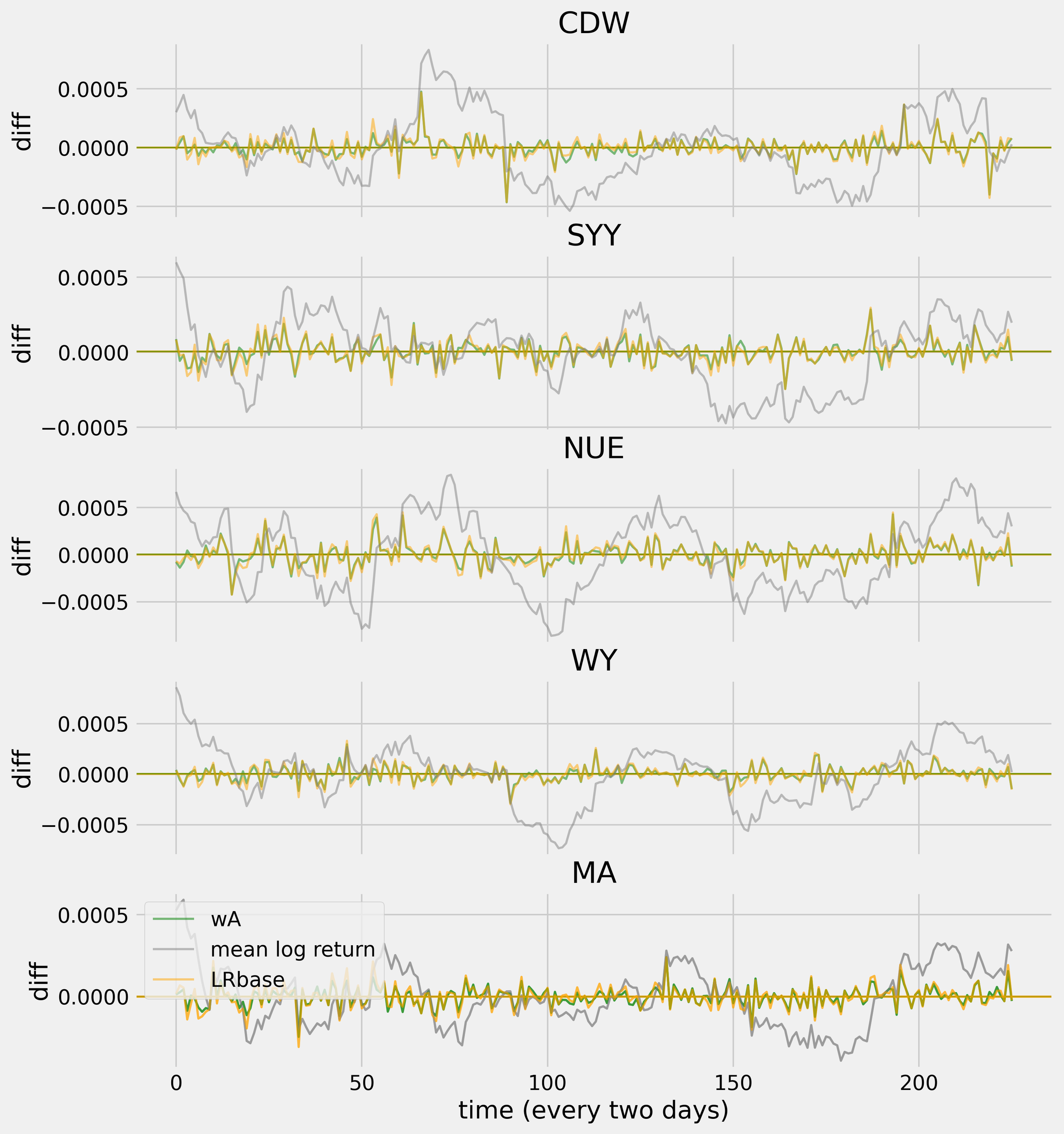

Supplement: S2 Fig — Differences between the wA prediction and the log return (green curves), the LRbase and the log return (orange curves) and the mean of the log return with itself (grey curves). The horizontal lines correspond to the mean of the differences. In this fugure, we show the difference of the predicted log return for the wA, the LRbase and the mean of the log return for 5 selected stock in the testing set. There is no qualitative difference between the LRbase prediction and the wA one. Both outperform a simple mean average of the log return. (TIF) [file pone.0319985.s002.tif]
