# Supplementary material for: Dynamical analysis of financial stocks network: Improving forecasting using network properties
Source: PLoS One. 2025 May 9;20(5):e0319985. doi: 10.1371/journal.pone.0319985 (PMC12063834; doi:10.1371/journal.pone.0319985)
Supplement: S1 Table — (PDF) [file pone.0319985.s003.pdf]

Table S1. Selected variables for the training of the long time period.

Rank	Variables	Correlation coefficients
26	Mean Closeness Centrality_3	0.09
25	Clustering_5	0.09
24	Max Eigenvalue Stock Returns_3	0.09
23	Mean Clustering_2	0.09
22	Resilience_5	0.09
21	Eigenvector Centrality_5	0.09
20	Resilience_2	0.09
19	Mean Eigenvector Centrality_4	0.09
18	Max Eigenvalue Stock Returns_2	0.10
17	Degree Centrality_4	0.10
16	90th Percentile Degree_4	0.10
15	90th Percentile Degree_5	0.10
14	Closeness Centrality_2	0.11
13	Degree Centrality_5	0.11
12	Resilience_1	0.11
11	Mean Eigenvector Centrality_5	0.11
10	Closeness Centrality_3	0.12
9	Resilience_4	0.12
8	Mean Closeness Centrality_5	0.13
7	Largest Component_5	0.13
6	Largest Component_2	0.15
5	90th Percentile Degree_2	0.15
4	Mean Closeness Centrality_2	0.17
3	90th Percentile Degree_1	0.22
2	Log Return_2	0.28
1	Log Return_1	0.63
