# Supplementary material for: Dynamical analysis of financial stocks network: Improving forecasting using network properties
Source: PLoS One. 2025 May 9;20(5):e0319985. doi: 10.1371/journal.pone.0319985 (PMC12063834; doi:10.1371/journal.pone.0319985)
Supplement: S2 Table — (PDF) [file pone.0319985.s004.pdf]

Table S2. 42 Selected variables over 63 for the training of the short time period: we don't show the ones with a correlation coefficient less than 0.10.

Rank	Variables	Correlation coefficients
42	Resilience_9	0.10
41	Largest Component_3	0.10
40	Max Eigenvalue Stock Returns_11	0.10
39	Resilience_8	0.11
38	Mean Closeness Centrality_3	0.11
37	90th Percentile Degree_4	0.11
36	Max Eigenvalue Stock Returns_10	0.11
35	Resilience_7	0.11
34	Max Eigenvalue Stock Returns_9	0.11
33	Max Eigenvalue Stock Returns_8	0.12
32	Largest Component_2	0.12
31	90th Percentile Degree_3	0.12
30	Mean Closeness Centrality_2	0.12
29	Resilience_6	0.12
28	Max Eigenvalue Stock Returns_7	0.13
27	Resilience_5	0.14
26	Mean Closeness Centrality_1	0.14
25	Max Eigenvalue Stock Returns_6	0.14
24	Largest Component_1	0.14
23	90th Percentile Degree_2	0.14
22	Max Eigenvalue Stock Returns_5	0.14
21	Resilience_4	0.15
20	Max Eigenvalue Stock Returns_4	0.15
19	90th Percentile Degree_1	0.15
18	Max Eigenvalue Stock Returns_3	0.16
17	Resilience_3	0.17
16	Max Eigenvalue Stock Returns_2	0.17
15	Max Eigenvalue Stock Returns_1	0.18
14	Resilience_2	0.18
13	Resilience_1	0.20
1 to 12	Log Return_1 to Log Return_12	0.95–0.44

The correlation coefficients in Tab.S1 and Tab.S2 show interesting properties. First, we find that the log return at previous lags weighs significantly more than the network variables for the prediction of the stock's log return. Then, we see that the network variable correlations are distributed from ~ 0.2 to ~ 0.1. Interestingly, in the short time period, the Resilience variable at lags 1, 2, and 3 weighs highly in the model, as well as the maximum eigenvalue of the stock returns. For the long time period, it is the 90th percentile of the degree at lags 1 and 2 that is consistently weighted as the top network feature variable.
